# Six‐transmembrane epithelial antigen of prostate 3 (STEAP3) is a potential prognostic biomarker in clear cell renal cell carcinoma that correlates with M2 macrophage infiltration and epithelial–mesenchymal

**DOI:** 10.1002/cnr2.1824

**Published:** 2023-06-21

**Authors:** Haotian Wei, Zhaochen Li, Yang Zhao, Shimiao Zhu, Simeng Wen, Changyi Quan

**Affiliations:** ^1^ Department of Urology Secord Affiliated Hospital of Tianjin Medical University Tianjin China; ^2^ Department of Radiology Secord Affiliated Hospital of Tianjin Medical University Tianjin China

**Keywords:** clear cell renal cell carcinoma, epithelial–mesenchymal transition, immune infiltration, M2 macrophage, prognostic biomarker, STEAP3

## Abstract

**Background:**

The six‐transmembrane epithelial antigen of the prostate 3 (STEAP3) is a metalloreductase, which is essential for iron uptake. Existing literature has shown that STEAP3 may perform an important role in the onset and progression of tumors. Nonetheless, a complete pan‐cancer investigation of the prognostic significance and immune properties of STEAP3 is currently unavailable.

**Aims:**

As part of our investigation into the possible functions of STEAP3 in malignancies, we conducted a comprehensive analysis to examine the prognostic value and immune features of STEAP3 in human pan‐cancer.

**Methods and Results:**

R and Cytoscape programs were applied to analyze and visualize the data. The expression of STEAP3 in both cell lines and tissues was measured utilizing a variety of approaches. Using the shRNA knockdown technique, we tested the viability of the A498 and 786‐O cell lines and validated their functions. Both CCK‐8 and transwell assays were conducted to estimate cell proliferation and invasion. The expression of STEAP3 was substantially elevated and was shown to be linked to prognosis in the majority of malignancies, notably in clear cell renal cell carcinoma (ccRCC). In addition, the expression of STEAP3 was shown to have a strong correlation with immune infiltrates, which in turn triggered the recruitment and polarization of M2 macrophages in ccRCC. The protein STEAP3 shows promise as a predictor of responses to immune‐checkpoint blockade (ICB) therapy. Positive links between STEAP3 and the epithelial‐mesenchymal transition (EMT), the p53 pathway, and the immune‐associated pathways were also found in the enrichment analysis. Our results illustrated that the STEAP3 expression level was substantially elevated in ccRCC tissues and suggested that it could stimulate EMT in ccRCC by downregulating CDH1.

**Conclusion:**

In a diverse range of cancers, STEAP3 might serve as a biomarker for determining the prognosis as well as a predictor of immunotherapy responsiveness. STEAP3 is a novel biological marker for determining prognosis, and it also performs a remarkable function in the promotion of tumor growth in ccRCC by enhancing invasion and EMT, as well as by triggering the recruitment and polarization of M2 macrophages.

## 1 INTRODUCTION

Cancer has a huge impact on human health worldwide. Cancer contributes to a large proportion of all deaths and ranks second as the leading contributor to mortality in the world after angiocardiopathy.^1^ Cancer therapy has rapidly progressed in the past decade. Immunotherapy, particularly immune‐checkpoint blockade (ICB), has emerged as a promising cancer treatment approach.^2^ However, a large population of patients fail to respond to immunotherapy or relapse, because of the heterogeneity of immune cell infiltrates. In light of this, the discovery of immune‐related biological markers that are both specific and sensitive continues to be an important step toward the development of innovative diagnostic and therapeutic techniques.

Iron homeostasis is crucial for maintaining cellular functions. Iron metabolism is closely related to cancer development^3^ and subtle changes can influence the local microenvironment and function of immune cells.^4^ Several researchers have reported the role of the six‐transmembrane epithelial antigen of the prostate (STEAP) family, a type of iron metabolism‐associated protein in different cancers.^5^, ^6^ STEAP family member 3 (STEAP3) performs a pivotal function in iron uptake as a metalloreductase by reducing ferric iron to ferrous iron.^7^, ^8^ STEAP3 may serve a remarkable role in tumorigenesis. It can promote hepatocellular carcinoma cell proliferation by enhancing RAC1–ERK–STAT3 signaling.^9^ Increased levels of STEAP3 expression are associated with a worse prognosis in a diverse variety of tumor types, including glioblastoma, breast carcinomas, and renal cell carcinoma.^10^, ^11^, ^12^ Further, STEAP3 may elicit specific cytotoxic lymphocyte responses and can be a promising candidate for prostate cancer immunotherapy.^13^ Nevertheless, a systematic review of STEAP3's predictive significance and immune features across all cancer types has not been documented.

Given STEAP3's specific functions in malignancies and its therapeutic potential, the prospects of its use as a pan‐cancer biomarker are particularly significant. Therefore, we conducted a systematic analysis of the prognostic value and immune characteristics of STEAP3. Furthermore, we performed pathway analysis to explore its regulation in cancer functional states and changes in the downstream signaling pathway. Subsequently, we constructed the competitive endogenous RNA (ceRNA) network that regulates STEAP3 expression in clear cell renal cell carcinoma (ccRCC) and validated the bioinformatic results through in vitro experiments. Our findings illustrated that STEAP3 is a new biological marker for ccRCC prognosis.

## 2 MATERIALS AND METHODS

### 2.1 Data access

From The Cancer Genome Atlas (TCGA) database (https://portal.gdc.cancer.gov/), the RNA‐seq data and relevant clinical data of 33 different types of cancer and normal tissues were obtained in June 2022.^14^ The ccRCC cohort comprised 530 ccRCC samples and 161 normal kidney tissues, which were used for further analyses. The number of patients in other cohorts and the TCGA cancer type abbreviations are summarized in Table S1.

We also obtained 946 cell lines expression matrix of tumors from the Cancer Cell Line Encyclopedia (CCLE; https://portals.broadinstitute.org/ccle) data set.^15^

The GSE15641 (Platform: GPL96) microarray data were obtained from the Gene Expression Omnibus (GEO) data set (http://www.ncbi.nlm.nih.gov/geo).^16^ We analyzed a total of 33 cases with ccRCC and 23 control kidney tissue samples. The unprocessed data were obtained in the format of MINiML files, and after extraction, they were subjected to log2 transformation to achieve normalization. The data obtained from the microarrays were normalized with the help of the function “normalize quantiles” found in the “preprocessCore” R package. Using the annotation information of the normalized data in the platform, the probes were transformed into gene symbols. Multiple‐gene‐matching probes were excluded from these sets of data. The average expression value of the gene measured by multiple probes was calculated as the final expression value.

### 2.2 Protein‐level analysis

The Human Protein Atlas (HPA) database (https://www.proteinatlas.org/) is one of the most frequently visited human proteome databases.^17^ We collected the STEAP3 expression data by analyzing the immunohistochemical (IHC) staining images obtained from the HPA database, and 190 samples were included for further analysis.

### 2.3 Differential expression analysis

The variances in STEAP3 expression levels between tumors and adjoining tissues were compared using R software (v3.4.1). A bar graph depicting STEAP3 expression was generated using the “ggplot2” R package. For a comparison between two groups, we utilized the Wilcoxon test, whereas, for a comparison among multiple groups, the Kruskal‐Wallis test was applied.

### 2.4 Survival prognosis analysis

Using the TCGA data set, we assessed STEAP3's prognostic significance for a subset of cancers and prognosis types via univariate Cox regression analysis by applying the “forestplot” R package, we visually represented the *p*‐value, hazard ratio (HR), and 95% confidence interval (CI) for each variable in a forest plot. Then, we applied Kaplan–Meier (KM) analysis using “survival” and “survminer” R packages to show the variation in overall survival (OS) between patients with high‐ and low‐expression groups to assess the prognostic significance of STEAP3. Finally, survival rates were compared among the groups using the Log‐rank test.

### 2.5 TISDB database

TISDB (http://cis.hku.hk/TISIDB/) is a web‐based platform that combines data from multiple sources. We used this database to determine whether STEAP3 expression is associated with pathological grades or clinical stages of ccRCC. We entered “STEAP3” in the “Quick Search” module, and links between STEAP3 and clinical features were obtained from the “Clinical” module.^18^

### 2.6 cBioPortal database

cBioPortal (http://www.cbioportal.org) is a usable and reliable portal to perform cancer genomics analysis. The mutational profile of STEAP3 in various tumors was analyzed using this database. “TCGA, PanCancer Atlas” was chosen to serve as the cohort. Thereafter, we entered “STEAP3” in the “Quick Search” module. The “cancer type summary” and the “mutation” modules both presented STEAP3 alteration sites, types, and numbers.^19^

### 2.7 Association of STEAP3 expression with tumor mutational burden and Microsatellite Instability

Tumor mutational burden (TMB) is defined as the count of somatic nonsynonymous mutations per megabase.^20^ Microsatellites are repeats of a few nucleotides (1–6 bp) that are distributed across the genome.^21^ We used TCGA‐derived RNA‐seq data to determine TMB and microsatellite instability (MSI) scores and then analyzed the relationships between TMB, MSI, and STEAP3 expression utilizing Spearman's correlation analysis.

### 2.8 Immune cell infiltration and immune checkpoint analyses

The reliability of the immune score assessment results was evaluated using the “immuneeconv” R package, which incorporates two latest algorithms: TIMER and CIBERSORT. Using TCGA‐derived data, we conducted a Spearman correlation analysis of the scores of 22 different immune cell subsets and STEAP3 expression.

Several transcripts, including SIGLEC15, TIGIT, CD274, HAVCR2, PDCD1, CTLA4, LAG3, and PDCD1LG2, have been linked to immune checkpoints. To determine the level of expression of genes relevant to immune checkpoints, we collected expression data of the above eight genes. Subsequently, using the information in the TCGA database, we implemented a Spearman correlation analysis to determine the link between the expression levels of the eight genes and STEAP3 expression.

### 2.9 Tumor immune syngeneic MOuse database analysis

Tumor immune syngeneic MOuse (TISMO) database is an immunotherapy‐response predictor database based on the syngeneic mouse tumor model. We used this database to access whether STEAP3 expression is related to immunotherapy response. We entered “STEAP3” in the “Gene” module and obtained results from the “Gene in Vivo” module,^22^ and 832 ICB studies were included in further analysis.

### 2.10 Correlation between STEAP3 and drug response

Gene set cancer analysis (GSCA) (http://bioinfo.life.hust.edu.cn/GSCA/#/) is an easy‐to‐use web server for the visualization of gene and drug sensitivity correlation. We used the GSCA database to examine the link between STEAP3 expression and sensitivity to drugs. We entered “STEAP3” in the “Drug” module. “GDSC drug sensitivity and expression correlation” and “CTRP drug sensitivity and expression correlation” were chosen for the correlation analysis.^23^ Additionally, the IC50 data for 732 small‐molecule drugs were included in further analysis.

### 2.11 Enrichment analysis of genes associated with STEAP3

STRING (https://cn.string-db.org/) is a network mapping tool based on the STRING protein interaction algorithm. Applying this database, we mapped out STEAP3's potential network of interaction, which was visualized using the Cytoscape software. We entered “STEAP3” in the “Protein by name” module. The main parameters that were established were as follows: network edge meaning (“evidence”), minimum threshold score for interaction [“low confidence (0.150)”], and a maximum number of interactors to show (“no more than 50 interactors” in the first shell).^24^

Enrichment analysis was conducted on the top 50 genes that were chosen based on the potential interaction network of STEAP3. These genes were searched in the Kyoto Encyclopedia of Genes and Genomes (KEGG) pathway and Gene Ontology (GO) database using the Metascape tool (http://metascape.org). We submitted the list of the top 50 genes on the home page and obtained the results in the “Analysis Report Page.”^25^

By using TCGA‐derived data, we used single sample gene set enrichment analysis (ssGSEA) to explore the pathways affected by STEAP3 with the “GSVA” R package. First, we obtained the Hallmark signatures from the Molecular Signatures Database.^26^ Afterward, we computed an enrichment score for each sample and conducted a Spearman correlation analysis of these scores and STEAP3 expression. Finally, the “ggplot2” R package presented the results in the form of a bubble plot, which included a summary of all the findings.

### 2.12 Single‐cell level pathway exploration

CancerSEA (biocc.hrbmu.edu.cn/CancerSEA/home.jsp) is a strong functional analysis software based on data obtained by sequencing single cells. In total, 93 475 cancer single cells from 74 single‐cell data sets were included for further analysis. We used this database to explore whether STEAP3 expression is related to 14 cancer‐associated functional states. We entered “STEAP3” in the “Search” module and obtained results from the “Relevance” module.^27^

### 2.13 Candidate ceRNA regulatory network prediction

The ceRNA regulatory network of STEAP3 was predicted using PITA, RNA22, miRmap, microT, miRanda, PicTar, TargetScan, and StarBase gene prediction programs.^28^ The relationship between miRNAs and lncRNAs was visualized in the Cytoscape tool.

### 2.14 Specimen collection

At the Second Hospital of Tianjin Medical University (Tianjin, China), we collected 10 pairs of ccRCC and relevant paracancerous tissues from patients who had had partial or radical nephrectomy from June to September 2022. All the patients were diagnosed with ccRCC based on the histopathological findings. Ethical approval was obtained from the Ethical committee of the Second Hospital of Tianjin Medical University. Every participant granted their written consent after receiving appropriate information. Table 1 provides a summary of the participant's clinical data from the trial.

**TABLE 1:** Clinical information of all patients.

Patient No.	Sex	Age (year)	Grade	T stage
1	Female	64	I	T1a
2	Male	71	II	T3a
3	Male	58	III	T3
4	Male	64	III	T1
5	Male	55	II	T1b
6	Male	59	III	T3
7	Male	73	III	T1b
8	Male	61	II	T1b
9	Male	59	II	T3
10	Male	74	II	T3a

### 2.15 RNA extraction and cDNA synthesis

To obtain total RNA from cells and tissues, we followed the directions provided by the manufacturer of the SPARKeasy bacterial/cell RNA kit (AC0202, Sparkjade). Thereafter, 2 the T series Multi‐Block thermal cycler (LongGene) was then utilized to synthesize first‐strand cDNA from 2 μg of total RNA.

### 2.16 Real‐time quantitative PCR

The STEAP3 relative mRNA expression was detected using Real‐time quantitative PCR (RT‐qPCR), with the fast start universal SYBR green master kits (S2008, BR Healthcare) and CFX96TM real‐time PCR System (Bio‐Rad). GADPH represented the endogenous normalization reference. All the primers are listed in Table 2.

**TABLE 2:** Primer's sequence used in real‐time quantitative PCR.

Gene	Primer sequence
STEAP3‐F	TGCAAACTCGCTCAACTGGAGG
STEAP3‐R	AGGCAGGTAGAACTTGTAGCGG
GADPH‐F	ACAACTTTGGTATCGTGGAAGG
GADPH‐R	GCCATCACGCCACAGTTTC
CDH2‐F	CCTCCAGAGTTTACTGCCATGAC
CDH2‐R	GTAGGATCTCCGCCACTGATTC
CDH1‐F	CGAGAGCTACACGTTCACGG
CDH1‐R	GGGTGTCGAGGGAAAAATAGG
VIM‐F	AGGCAAAGCAGGAGTCCACTGA
VIM‐R	ATCTGGCGTTCCAGGGACTCAT

### 2.17 Cell culture and antibody

Tianjin Institute of Urology (Tianjin, China) provided the 786‐O and A498 cell lines. Afterward, cells were cultured in RPMI‐1640 (C11875500BT, Gibco) with 10% fetal bovine serum (04‐001‐1A, Biological Industries) and 1% penicillin–streptomycin (C7072, Bioss) at 37°C.

The primary anti‐STEAP3 (PA5‐62202, Thermo Scientific) and anti‐alpha‐tubulin (A11126, Thermo Scientific) rabbit antibodies were diluted following the manufacturer's specifications.

### 2.18 Western blotting

The RIPA buffer was utilized for the total protein extraction. The proteins were separated using SDS‐PAGE (10% resolving gel) before transferring them onto PVDF membranes. The next step involved incubating the membranes for an entire night with diluted primary antibodies followed by 30 min of incubation with the secondary antibody. The gel imaging system (Tianneng) was used to detect the signal strength.

### 2.19 Immunohistochemical analysis

Paraffin blocks of tumor tissues were used to prepare unstained slides for immunohistochemistry. In brief, paraffin sections were immunostained using a streptavidin peroxidase procedure after heat repair of the antigen. The signal was detected using a 3,3′‐diaminobenzidine solution and hematoxylin and the slides were observed using a microscope (Leica, Germany).

### 2.20 shRNA and lentivirus transduction

Specific shRNA against STEAP3 (GCUUCAUGCCCGUGGACAUTT) and its corresponding control were purchased from JiKai Gene. shSTEAP3 was transduced into 786‐O and A498 cells, and puromycin was used to select cells showing stable transfection for 2 weeks. The detailed procedures were mentioned in the operation manual provided by JiKai Gene.

### 2.21 Cell proliferation assay

In 96‐well plates (Corning), cell seeding was conducted at a density of 3000 cells per well. and cultured for 0, 24, 72, and 120 h. Thereafter, each well was supplied with 10 μL CCK‐8 reagent (Beyotime) and subjected to incubation for 2 h at 37°C. The microplate reader (Bioteck) was utilized to measure the absorbance at 450 nm.

### 2.22 Cell migration assay

We added 20 000 cells with 200 μL serum‐free medium (SFM) in the upper chamber and 600 μL complete medium in the lower chamber of 24‐well plates (Corning). After 16 h, the cells were stained with 1% crystal violet for 5 min after being fixed for 20 min with 4% tissue fixative. Lastly, the migrated cells were observed using a microscope (Leica).

### 2.23 Statistical analysis

Both SPSS 23 and R software v4.03 were utilized in the analyses of statistical data. Table S2 details the R packages that were employed in this investigation. Analysis of STEAP3 expression levels was conducted utilizing the Wilcoxon test for two‐group data, whereas the Kruskal‐Wallis test was conducted for multiple‐group data to test statistical significance. Expression profiles of STEAP3 were evaluated by comparing clinical ccRCC samples and adjoining normal tissues using the paired *t* test for evaluating statistical significance. The predictive significance of STEAP3 was determined using univariate Cox regression analysis and KM curves. For KM curves, *p*‐values, and HR with 95% CI were generated by log‐rank tests and univariate cox proportional hazards regression. To assess the statistical correlations between STEAP3 and other variables, we conducted a Spearman correlation analysis. P‐values of less than 0.05 indicated a significance level.

## 3 RESULTS

### 3.1 Pan‐cancer analysis of STEAP3 expression

**FIGURE 1:**
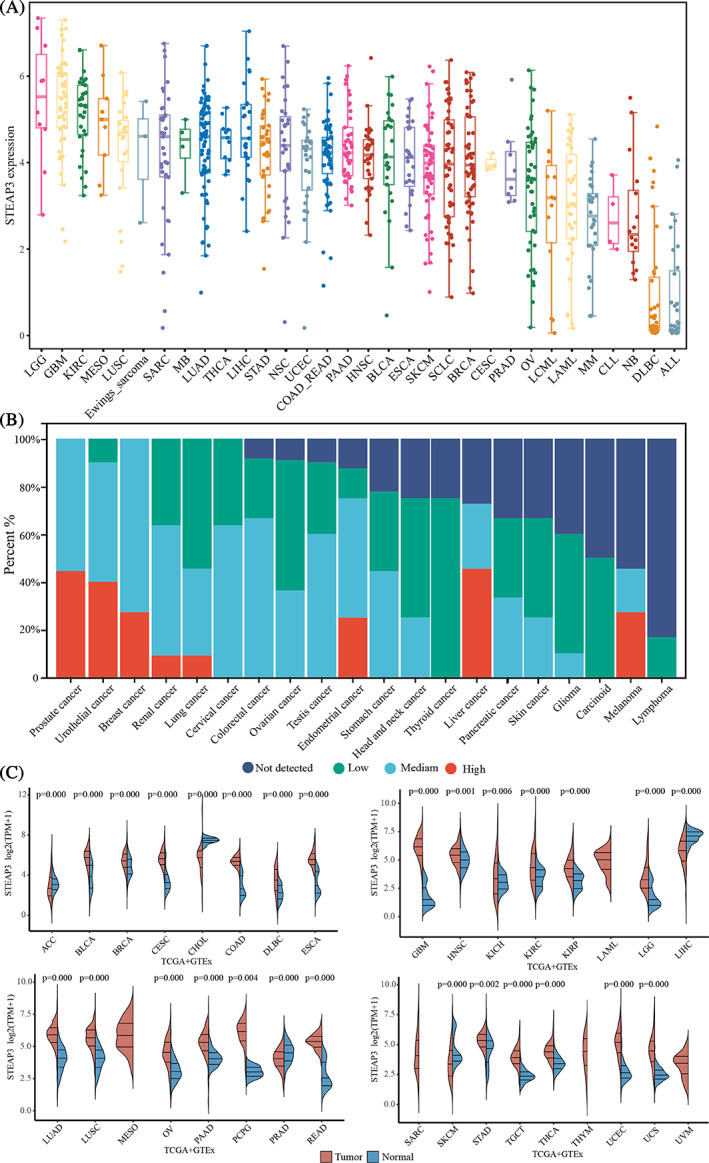
The expression landscape of STEAP3 in human pan‐cancer. (A) The distribution of mRNA expression of STEAP3 in distinct cell lines based on CCLE database. (B) The protein expression distribution of STEAP3 by immunohistochemistry in different cancers based on HPA database. (C) The mRNA expression distribution of STEAP3 in tumor tissues and normal tissues based on TCGA database. The Wilcoxon test was utilized to examine the significance of variations between two sets of data.

To gain more insights into STEAP3's expression profile, we evaluated its mRNA expression in 946 tumor cell lines from the CCLE database. According to the mean expression values, GBM, LGG, and KIRC ranked as the top three cancers. DLBC and ALL exhibit the lowest expression (Figure 1A). Then, we measured the protein expression of STEAP3 in various cancers in the HPA database. IHC data of 190 samples revealed that STEAP3 is highly expressed in lung, renal, urothelial, prostate, breast, and liver cancers (Figure 1B). We subsequently made a comparison in STEAP3 expression between tumors and normal samples to probe how STEAP3 exerts its oncogenic activity. Twenty‐two different types of cancer showed remarkably elevated STEAP3 expression in contrast with normal tissues: UCS, UCEC, THCA, TGCT, STAD, READ, PCPG, PAAD, OV, LUSC, LUAD, LGG, KRIP, KIRC, HNSC, GBM, ESCA, DLBC, COAD, CESC, BRCA, and BLCA Conversely, STEAP3 were considerably downregulated in five cancer types, namely ACC, CHOL, LIHC, PRAD, and SKCM (Figure 1C). The additional details are included in Table S3.

### 3.2 Prognostic values of STEAP3

**FIGURE 2:**
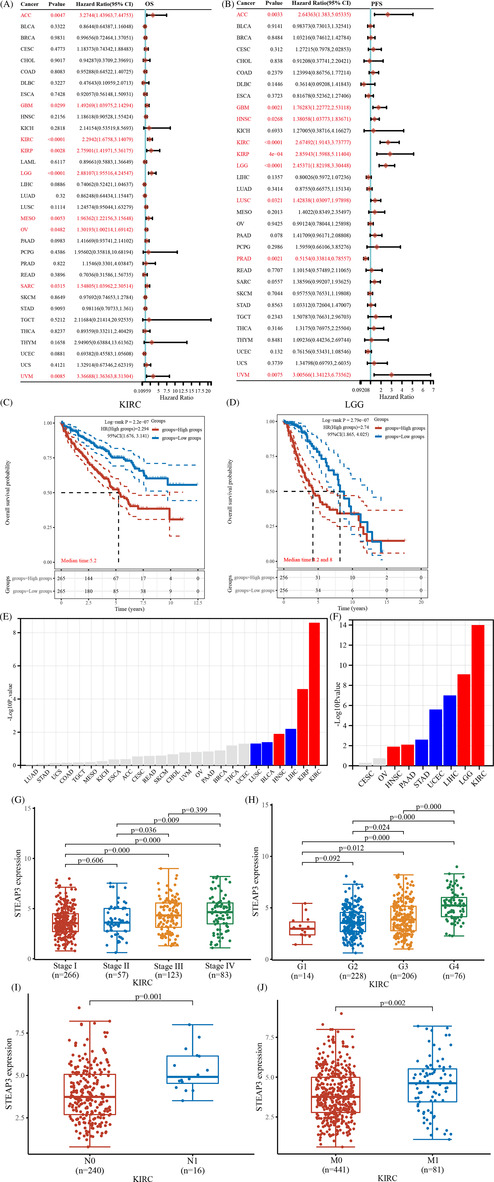
Association between STEAP3 expression and prognosis in pan‐cancer. (A, B) The predictive significance of STEAP3 in various malignancies was shown in a forest plot using univariate Cox regression. (C, D) Kaplan–Meier overall survival curves of STEAP3 in ACC, KIRC, KIRP, and LGG. (E) The correlations between the STEAP3 expression and tumor clinical stage. Red represents a positive and blue a negative correlation. Statistical correlations were assessed using a Spearman correlation analysis. (F) The correlations between the STEAP3 expression and tumor pathological stage. Red represents a positive and blue a negative correlation. Statistical correlations were assessed using a Spearman correlation analysis. (G) The expression distribution of STEAP3 in the different clinical stages. STEAP3 expression levels in Stage I, II, III, and IV groups were 3.69 ± 1.41, 3.80 ± 1.56, 4.34 ± 1.66, and 4.55 ± 1.74. Wilcoxon test was employed to examine the significance of differences between two sets of data. (H) The expression distribution of STEAP3 in the different pathological grades. STEAP3 expression levels in Grade 1, 2, 3, and 4 groups were 3.12 ± 1.07, 3.65 ± 1.35, 3.98 ± 1.63, and 5.24 ± 1.44. Wilcoxon test was employed to examine the significance of differences between two sets of data. (I) The expression distribution of STEAP3 in the different N stages. STEAP3 expression levels in the N0 and N1 groups were 3.94 ± 1.59 and 5.31 ± 1.23. Wilcoxon test was employed to examine the significance of differences between two sets of data. (J) The expression distribution of STEAP3 in the different M stages. STEAP3 expression levels in the M0 and M1 groups were 3.91 ± 1.53 and 4.52 ± 1.73. Wilcoxon test was employed to examine the significance of differences between two sets of data.

We conducted a survival analysis for STEAP3 in pan‐cancer. In patients with UVM, GBM, KIRC, LGG, MESO, KIRP, OV, SARC, and ACC, STEAP3 was a risk factor for unfavorable OS (Figure 2A). STEAP3 was also a risk factor for the progression‐free survival (PFS) of patients with UVM, LUSC, LGG, KIRP, KIRC, HNSC, GBM, and ACC. However, among those with PRAD, STEAP3 exerted a protective function (Figure 2B). We analyzed OS and PFS values and determined the prognostic significance of STEAP3 through KM analysis. Our data illustrated that the elevated expression level of STEAP3 in ACC, KIRC, KIRP, and LGG was linked to worse survival status (Figure 2C,D and Figure S1A,B). We subsequently examined STEAP3 expression in various stages using the TISDB database to better understand its clinical relevance. According to the findings, STEAP3 was expressed at a higher level in KIRP, HNSC, and KIRC at the advanced clinical stage but at a lower level in LIHC, BLCA, and LUSC (Figure 2E). Similarly, we found that STEAP3 was expressed at a higher level in the advanced pathological grade of KIRC, LGG, PAAD, and HNSC and lowly expressed in the advanced pathological grade of LIHC, USEC, and STAD (Figures 2F). Particularly in KIRC, there was an upward trend in STEAP3 expression with increasing tumor stage and pathological grade (Figure 2G,H). Notably, our results showed that STEAP3 was expressed at a higher level in ccRCC individuals with lymph node and distance metastases (Figure 2I,J).

### 3.3 The characteristics of STEAP3 mutations

**FIGURE 3:**
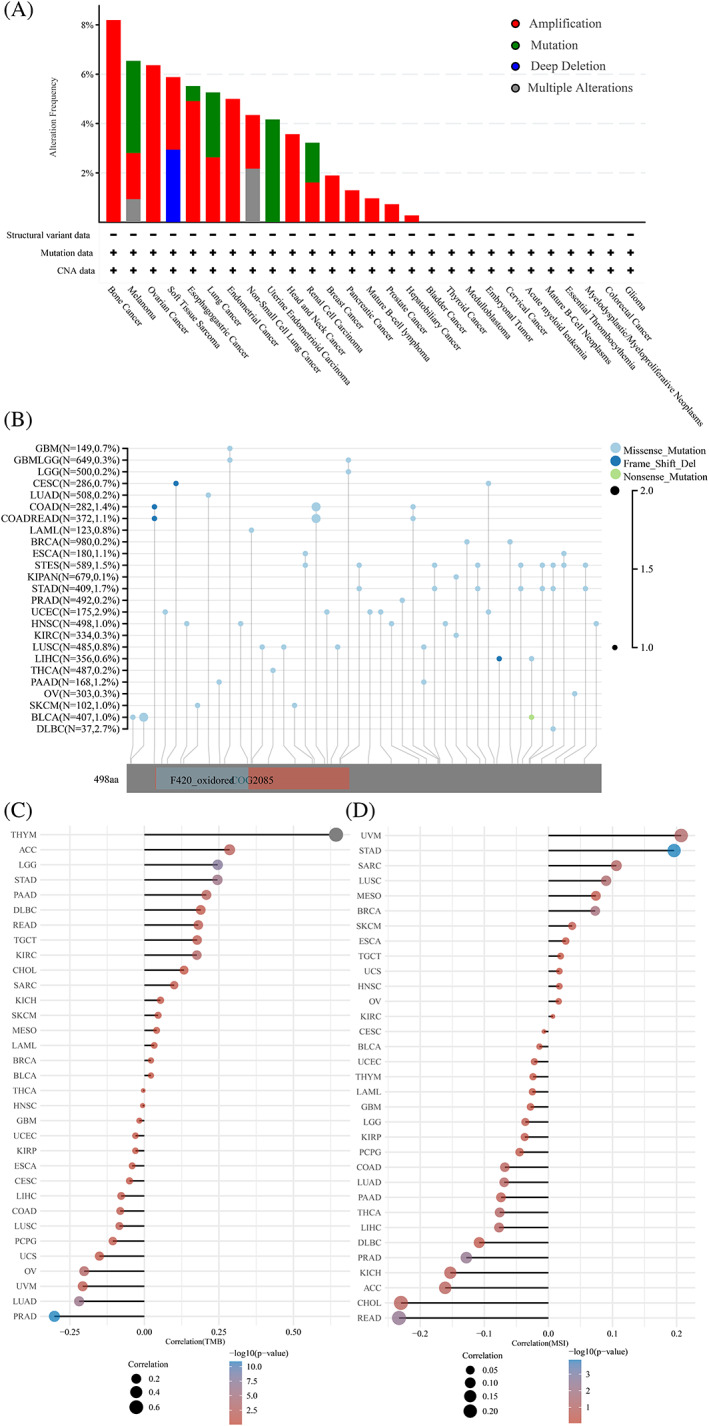
Analysis of STEAP3 gene mutation in pan‐cancer. (A) The STEAP3 gene alteration frequency with different types of mutations in pan‐cancer. (B) Mutation diagram of STEAP3 in pan‐cancer across protein domains. (C, D) Pan‐cancer analysis of the correlation between STEAP3 expression and immunomodulators TMB and MSI. Statistical correlations were assessed using a Spearman correlation analysis.

Considering the abnormal STEAP3 expression observed in different cancers, we analyzed mutations in the STEAP3 genes. We found that the highest alteration frequency (>8%) appeared in bone cancer. The alterations of STEAP3 were uncommon, and amplification of the STEAP3 gene was the most prevalent type of genetic modification, followed by mutation (Figure 3A). cBioPortal database analysis showed that missense mutations were the main type of STEAP3 gene mutations in pan‐cancer (Figure 3B). TMB and MSI are the potential predictors of the response to ICB therapy.^29^ STEAP3 expression and TMB were positively correlated in THYM, LGG, STAD, KIRC, PAAD, ACC, READ, and TGCT and negatively correlated in PRAD, LUAD, and OV (Figure 3C). STEAP3 expression and MSI were positively correlated in STAD, BRCA, and LUSC and negatively correlated in READ and PRAD (Figure 3D).

### 3.4 Pan‐cancer analysis of STEAP3 expression and immune cell infiltration

**FIGURE 4:**
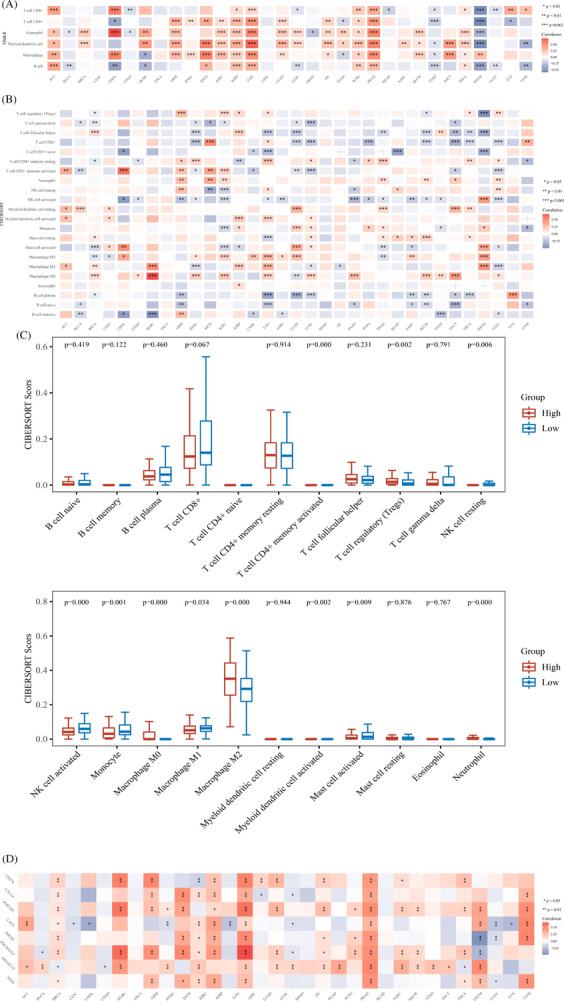
Correlation of STEAP3 with immune cell infiltration and immune checkpoint genes in pan‐cancer. (A, B) The correlations between the STEAP3 expression and the infiltration levels of various immune cells based on the TIMER and CIBERSORT algorithms. Statistical correlations were assessed using a Spearman correlation analysis. (C) The difference of immune microenvironment between the high STEAP3 expression group and low STEAP3 expression group in KIRC. Wilcoxon test was employed to examine the significance of differences between two sets of data. (D) The correlations between the STEAP3 and the immune checkpoints related genes expression. Statistical correlations were assessed using a Spearman correlation analysis.

We evaluated the correlations between STEAP3 expression and immune cell infiltrations premised on the TIMER algorithms. As per the findings, there was a strong correlation between STEAP3 expression and the degree of infiltration of most immune cells in pan‐cancer. THYM and STAD had an inverse correlation with STEAP3, whereas PRAD, KICH, PCPG, KIRP, LGG, LUAD, KIRC, GBM, ACC, OV, and THCA showed a positive link to STEAP3 (Figure 4A). We sorted immune cells using CIBERSOR algorithms to further identify the association of STEAP3 expression with immune cell infiltration levels. The results were significantly different for various cancer types (Figure 4B). Notably, STEAP3 expression was strongly linked to the abundance of Tregs, M0 and M2 macrophages, NK cell activated, monocytes, NK cell resting, mast cell activated, neutrophils, and T cell CD4+ memory‐activated in ccRCC. Notably, STEAP3 expression showed a positive link to the levels of M2 macrophages but not to that of M1 macrophages. We classified two groups as per the STEAP3 expression (low and high) in the TCGA platform to further identify the impact of STEAP3 on immune cell infiltrates. Then, we compared the difference in immune microenvironment between the two groups (Figure 4C). The high STEAP3 expression group was shown to have higher infiltrated levels of M2 macrophages and lower M1 macrophages compared with the group with low STEAP3 expression. Further investigation was done into the link between the expression of STEAP3 and eight common immune checkpoint genes (ICGs). STEAP3 expression has a positive correlation with the expression of ICGs in a majority of cancers, particularly in PRAD, GBM, LGG, KIRP, KIRC, and KICH (Figure 4D).

### 3.5 STEAP3 and the response to cancer therapy

**FIGURE 5:**
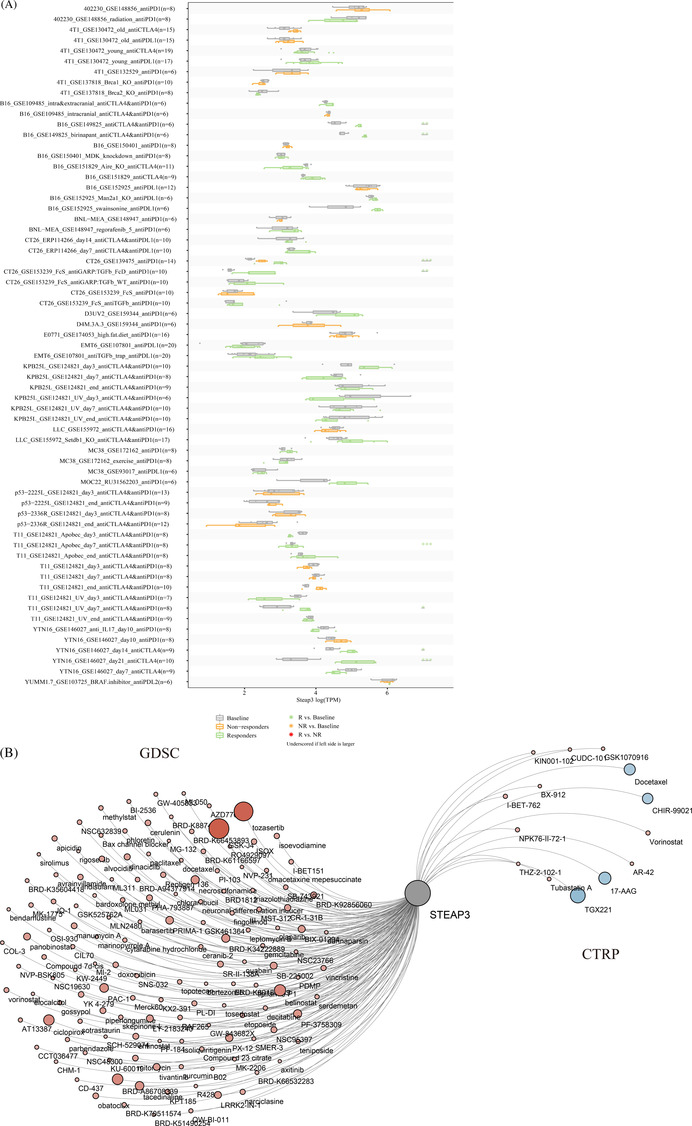
STEAP3 predicts the response to immunotherapy, chemotherapy agents, and small molecular compounds. (A) STEAP3 expression before and after immune‐checkpoint blockade treatments in different cancers. The asterisk (*p) represents the degree of importance (*p < .05, **p < .01, ***p < .001). (B) The correlations between the STEAP3 expression and drug IC50 based on GDSC and CTRP database. Statistical correlations were assessed using a Spearman correlation analysis.

We further analyzed the predictive role of STEAP3 in ICB therapy. We identified the expression of STEAP3 after immunotherapy using the TISMO database. 832 ICB studies were included for further analysis. The results showed that the STEAP3 expression levels in different models were significantly upregulated in the responders but not in the non‐responders after ICB therapy (especially anti‐CTLA4 antibody treatment), which implied that the upregulated STEAP3 was related to a better response for ICB therapy (Figure 5A). The additional details are included in Table S4. Therefore, the potential prediction ability of STEAP3 was further confirmed in the ICB response.

Further, we analyzed the relationships between STEAP3 expression and 732 common chemotherapy agents and small molecular compounds. Combining mRNA expression and drug sensitivity data from GDSC and CTRP, correlation analysis was conducted to find sensitive drugs (Figure 5B). We did not find any significantly high correlation (*r* > .5) between STEAP3 expression and drug IC50 in most cases. GSK‐J4 and tozasertib were the only two drugs having a significantly high correlation with STEAP3 expression. The additional details are shown included in Table S5.

### 3.6 Molecular function analysis of STEAP3 in pan‐cancer

**FIGURE 6:**
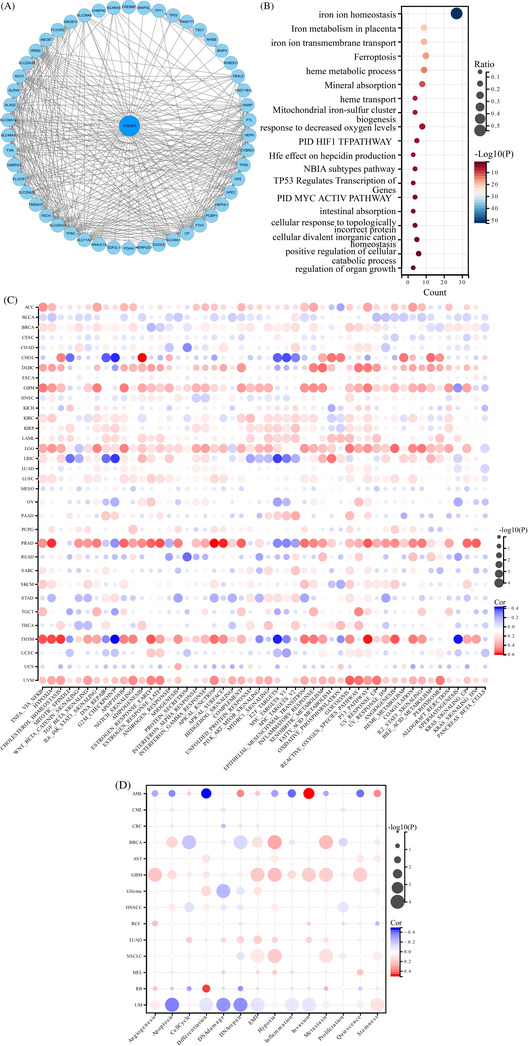
Protein–protein interaction network and functional enrichment of STEAP3. (A) The protein–protein interaction network of STEAP3 based on the STRING database. (B) GO and KEGG functional enrichment of STEAP3‐related genes. (C) The hallmarks ssGSEA of STEAP3 in pan‐cancer. The size of the circle represents the *p*‐value of each cancer enrichment item, and the color represents the *r*‐value of each enrichment item. Statistical correlations were assessed using a Spearman correlation analysis. (D) The correlation between STEAP3 expression and 14 cancer functional states using single‐cell sequence data from the CancerSEA database. The size of the circle represents the *p*‐value of each cancer enrichment item, and the color represents the *r*‐value of each enrichment item. Statistical correlations were assessed using a Spearman correlation analysis.

The 50 most correlated genes were obtained from the STRING database to further analyze the functional characteristics of STEAP3, and the potential interaction network was constructed (Figure 6A). GO/KEGG enrichment analysis showed that most related terms correlated with iron metabolism (Figure 6B). Next, we analyzed functional characteristics based on the hallmark terms (based on the ssGSEA score as the standard). We discovered a positive link between STEAP3 and immune‐associated pathways in diverse cancers, particularly in PRAD, ACC, LGG, KIRC, LIHC, LUSC, GBM, and THYM. We also found that p53 pathways and EMT were positively related to STEAP3 (Figure 6C). Then, utilizing single‐cell sequencing data from the CancerSEA database, we investigated the correlation between STEAP3 expression and 14 cancer functional states, and 93 475 cancer single cells from 74 single‐cell data sets were included for further analysis. STEAP3 was positively related to hypoxia, EMT, invasion, metastasis, and stemness in most tumors (Figure 6D). For ccRCC, a strong positive link between STEAP3 expression and hypoxia and stemness was observed, and a negative correlation with DNA repair and inflammation was observed. We explored the upstream ncRNA regulatory network of STEAP3 to investigate the unknown role of STEAP3. Forty‐five candidate miRNAs and 56 lncRNAs were predicted using various prediction programs (Figure S2A,C). Considering expression, survival, and correlation analyses, the most potential ceRNA networks may be SNHG4/hsa‐miR‐204‐5p/STEAP3 axis and LINC00997 or CYTOR/hsa‐miR‐27b‐3p/STEAP3 axis in ccRCC (Figure S2B,D).

### 3.7 STEAP3 promotes invasion and EMT of ccRCC

**FIGURE 7:**
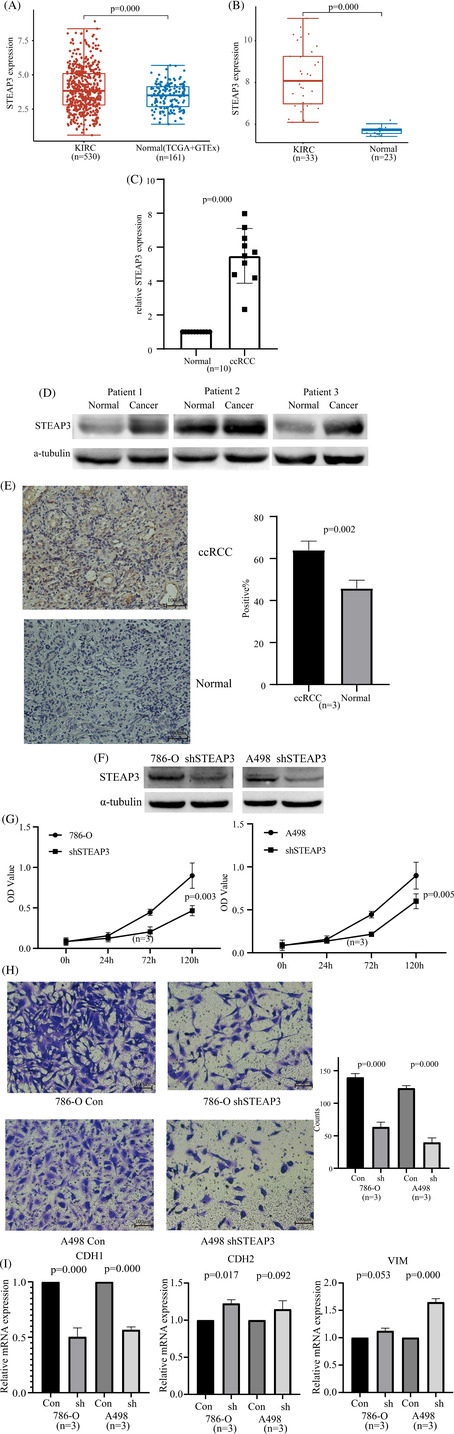
The expressions and biological functions of STEAP3 in ccRCC. (A) STEAP3 expression level in normal renal tissue and renal clear cell cancer tissue from TCGA database. STEAP3 expression levels in the KIRC and Normal groups were 3.97 ± 1.57 and 3.44 ± 0.91. The Wilcoxon test was utilized to examine the significance of variations between two sets of data. (B) STEAP3 expression level in normal renal tissue and renal clear cell cancer tissue from GSE15641 database. STEAP3 expression levels in the KIRC and Normal groups were 8.24 ± 1.43 and 5.69 ± 0.19. The Wilcoxon test was utilized to examine the significance of variations between two sets of data. (C) QRT‐PCR analysis of STEAP3 expression in 10 pairs of ccRCC and normal tissues. Gene expression values were normalized to GADPH expression values. Normal group was used as control. STEAP3 relative expression level in the ccRCC groups was 5.49 ± 1.62 (*n* = 10). The significant difference of two paired‐group data was tested with the paired *t* test. (D) Expression of STEAP3 detected in paired ccRCC and adjacent normal tissues using western blot (*n* = 3). α‐tubulin is included as a reference gene. (E) Representative images of immunohistochemistry showing STEAP3 expression in paired ccRCC and adjacent normal tissues. STEAP3 relative expression levels in the ccRCC and normal groups were 63.98 ± 4.29 and 45.78 ± 3.93 (*n* = 3). The significance difference of two paired‐group data was tested with the paired *t* test. (F) Expression of STEAP3 detected after transfection with shRNA in 786‐O and A498 cell lines using western blot. α‐tubulin is included as a reference gene. (G) The CCK‐8 assay indicated that the knockdown of STEAP3 weakened the proliferation ability of 786‐O and A498 cell lines. OD values in the 786‐O and ShSTEAP3 groups were 0.89 ± 0.06 and 0.47 ± 0.03 (*n* = 3). OD values in the A498 and ShSTEAP3 groups were 0.87 ± 0.04 and 0.60 ± 0.04 (*n* = 3). The significant difference of two paired‐group data was tested with the paired *t* test. (H) The transwell experiments indicated that the knockdown of STEAP3 weakened the invasive ability of 786‐O and A498 cell lines. Counts in the 786‐O and ShSTEAP3 groups were 139.67 ± 5.69 and 63.33 ± 7.57 (*n* = 3). Counts in the A498 and ShSTEAP3 groups were 123.00 ± 4.00 and 39.67 ± 7.02 (*n* = 3). The significance difference of two paired‐group data was tested with the paired *t* test. (I) RT‐qPCR analysis of EMT marker (CDH1, CDH2, VIM). Gene expression values were normalized to GADPH expression values. 786‐O and A498 groups were used as control. CDH1 relative expression level in the 786‐O shSTEAP3 and A498 shSTEAP3 groups was 0.50 ± 0.08 (*n* = 3) and 0.56 ± 0.03 (*n* = 3). CDH2 relative expression level in the 786‐O shSTEAP3 and A498 shSTEAP3 groups was 1.22 ± 0.05 (*n* = 3) and 1.14 ± 0.12 (*n* = 3). VIM relative expression level in the 786‐O shSTEAP3 and A498 shSTEAP3 groups was 1.12 ± 0.05 (*n* = 3) and 1.65 ± 0.06 (*n* = 3). The significant difference of two paired‐group data was tested with the paired *t* test.

STEAP3 mRNA levels were elevated in the ccRCC tumor tissues relative to the normal tissues according to the TCGA and GSE15641 databases (Figure 7A,B). These results were confirmed via RT‐qPCR, western blotting, and immunohistochemistry. We discovered that STEAP3 was expressed at a high level in patients with ccRCC (Figure 7C–E). Next, we confirmed our results through in vitro experiments. We used specific STEAP3‐targeting shRNAs to knock down STEAP3 in the 786‐O and A498 cells. STEAP3 knockdown was confirmed using western blotting. The results of CCK‐8 and transwell assays demonstrated that the knockdown of STEAP3 inhibited the proliferation and invasion of the 786‐O and A498 cells (Figure 7G,H). Thereafter, changes in the expression of EMT marker genes (CDH1, CDH2, and VIM) in 786‐O and A498 cells before and after STEAP3 knockdown were detected using RT‐qPCR. We found that STEAP3 knockdown significantly lowered CDH1 levels and elevated CDH2/VIM levels in 786‐O and A498 cells (Figure 7I).

## 4 DISCUSSION

Dysregulation of iron metabolism is closely related to cancer development. Recent studies on new proteins involved in iron metabolism have shed light on the interaction between iron and cancer,^30^, ^31^, ^32^ and these studies also suggest that proteins involved in iron metabolism may be multifunctional and contribute to the development of tumors through non‐iron metabolic pathways.^33^ Iron is an important factor in EMT, metastasis, and immunomodulation in human cancers.^4^, ^34^, ^35^ STEAP3, an iron reductase, was associated with the infiltration of immune cells in hepatocellular carcinoma and induced PD‐L2 expression.^36^ It may induce the formation of a tumor immune microenvironment in ccRCC.^37^ STEAP3 may play a role in cancer development, progression, and immunotherapy; however, few studies have explored its significance in cancer research.

First, we assessed STEAP3 expression in pan‐cancer cells. Data indicated that STEAP3 is relatively highly expressed in lung, renal, urothelial, prostate, breast, and liver cancers based on the HPA database. Compared with normal tissues, STEAP3 is significantly upregulated in 22 cancer types in TCGA database, suggesting that STEAP3 may function as a key regulator in cancers. Next, we examined the prognostic value of STEAP3. Analyses of patients' survival status confirmed that STEAP3 was a risk factor in the onset and progression of many different types of cancer. In ccRCC, STEAP3 was expressed at high levels in tumor tissues suggesting poor prognosis and correlated significantly with both clinical stages and pathological grades. Moreover, we discovered that STEAP3 was expressed at high level in ccRCC with lymph nodes and distant metastasis. Taken together, STEAP3 may be involved in tumor metastasis in ccRCC. DNA mutation is one of the critical factors during oncogenesis.^38^ However, we found that low frequency of alteration in the STEAP3 gene. This may because the highly conserved nature of pathways of iron metabolism.

Some researchers reported that STEAP3 are naturally processed CTL epitopes possessing anti‐prostate cancer reactivity in vivo and exhibited great potential for immunotherapy.^13^ In most malignancies, we observed a positive link between STEAP3 expression and the abundance of immune cells. The proportion of M2 macrophages in ccRCC was strongly linked to the level of STEAP3 expression, although this was not the case for M1 macrophages. Infiltration levels of M2 macrophages were greater whereas those of M1 macrophages were lower in the high STEAP3 expression group. We suggest that STEAP3 exerts its promoter functions in ccRCC by inducing the recruitment and polarization of M2 macrophages. We also verified that STEAP3 expression is intimately linked to MSI, TMB, and immune checkpoint gene expression, which are good potential markers for immunotherapy. STEAP3 expression levels in the different models are significantly upregulated in the responders after ICB therapy, indicating that it may be a promising immunotherapy response predictor. Moreover, we predicted GSK‐J4 and tozasertib as the sensitive drugs based on STEAP3 expression. GSK‐J4 is a small‐molecule inhibitor of histone 3 lysine 27 demethylation, which exerts anticancer effects in various tumors and has anti‐inflammatory effects.^39^ Tozasertib (MK‐0457) is a pan‐aurora kinase inhibitor, which suppresses cell proliferation and induces apoptosis in both preclinical studies and clinical settings.^40^ These results might lead to novel strategies for immunotherapy and chemotherapy.

We performed a functional analysis of STEAP3 in pan‐cancer and found that STEAP3 was strongly linked to immune‐related pathways, p53 pathways, and EMT in addition to iron metabolism. This should not be so surprising because STEAP3 encodes a multipass membrane protein that functions as an iron transporter. Based on single‐cell RNA‐sequencing data, we noted that STEAP3 was favorably linked to hypoxia, EMT, invasion, metastasis, and stemness in most tumors. The above findings confirmed that STEAP3 expression was linked to the activation of the immune‐related pathways and promoted tumorigenesis and progression by p53 pathways and EMT. Multiple studies have also corroborated our results and reported that STEAP3 is closely associated with the p53 signaling pathway.^41^, ^42^

Extensive research has demonstrated the significant involvement ncRNAs in tumorigenesis by competing for endogenous RNA.^43^, ^44^, ^45^ We predicted SNHG4/hsa‐miR‐204‐5p /STEAP3 axis and LINC00997 or CYTOR/hsa‐miR‐27b‐3p/STEAP3 axis as the most potential ceRNA networks in ccRCC. The role of these miRNAs and lncRNAs in other cancers also suggests their possible role in ccRCC. Hsa‐miR‐204‐5p and hsa‐miR‐27b‐3p perform an anticancer role in other cancer types. Laryngeal squamous cell carcinoma invasion and metastasis may be inhibited by Hsa‐miR‐204‐5p.^46^ Hsa‐miR‐27b‐3p can inhibit invasion, migration and EMT in gastric cancer^47^ and suppress glioma development via targeting YAP1.^48^ SNHG4 sponges miR‐204‐5p and promotes RCC cell proliferation and invasion.^49^ The lncRNA CYTOR performs a remarkable function in promoting the development of digestive system tumors.^50^, ^51^, ^52^ LINC00977 acts as an oncogene by binding STAT3 in ccRCC.^53^ Furthermore, by sponging miR‐512‐3p, LINC00997 is successful in suppressing the onset and progression of colorectal cancer.^54^

We further performed experiments to partially validate the analysis results in ccRCC. First, we confirmed STEAP3 expression levels in different patients using several methods. We performed a transwell assay and RT‐qPCR to check the effects of STEAP3 on EMT and speculated that STEAP3 stimulated the invasion and EMT through the downregulation of CDH1.

Nonetheless, there are several drawbacks to our investigation. First, the clinical sample size was relatively small and may not be sufficient to fully describe the problem. Second, the immune infiltration‐related analysis was predicted by algorithms based on RNA‐seq data, and we could not obtain the real immune cell infiltration data. Although we verified the results obtained from bioinformatic analysis using in vitro experiments as much as possible, more data are required. However, despite these limitations, we still obtained partially reliable conclusions, and we will further validate the available results and explore possible mechanisms in subsequent studies.

## 5 CONCLUSIONS

Herein, we conducted an in‐depth pan‐cancer investigation of STEAP3's role in cancer prognosis and immunology, demonstrating that STEAP3 could potentially serve as a predictive biological marker as well as a predictor of the responsiveness to immunotherapy in a variety of malignancies. STEAP3 is a new prognostic biomarker for ccRCC and exerts tumor‐promoting function via stimulating the invasion and EMT and inducing recruitment and polarization of M2 macrophages. Nonetheless, additional research is necessary to investigate the associated molecular pathways.

## AUTHOR CONTRIBUTIONS

**Haotian Wei:** Formal analysis (lead); investigation (equal); visualization (lead); writing – original draft (equal); writing – review and editing (equal). **Zhaochen Li:** Investigation (equal); writing – original draft (equal). **Yang Zhao:** Resources (equal). **Shimiao Zhu:** Conceptualization (equal); resources (equal); writing – review and editing (equal). **Simeng Wen:** Conceptualization (equal); writing – review and editing (lead). **Changyi Quan:** Conceptualization (lead); supervision (lead).

## CONFLICT OF INTEREST STATEMENT

The authors declare that the research was conducted in the absence of any commercial or financial relationships that could be construed as a potential conflict of interest.

## ETHICS STATEMENT

The Ethics Committee of the second hospital of Tianjin Medical University granted its approval to the patient sample research (ky2019k036). Every patient granted their written consent after receiving appropriate information.

## Supporting information


**Supplementary Figure S1.** Association between STEAP3 expression and prognosis in pan‐cancer.
**(A‐B)** Kaplan–Meier overall survival curves of STEAP3 in ACC and KIRP.
**(C)** The expression distribution of STEAP3 in the different clinical stages of HNSC, KIRP, LIHC, and LUSC.
**(D)** The expression distribution of STEAP3 in the different pathological stages of HNSC, LGG, LIHC, PAAD, STAD, and USEC.
**Supplementary Figure 2.** The lncRNA‐miRNA‐STEAP3 regulatory network in ccRCC.
**(A)** Upstream miRNAs that could potentially bind to STEAP3. The red circle represents positive correlation, the blue circle represents negative correlation, and the size of the circle represents the absolute value of the correlation coefficient, the larger the absolute value the larger the circle.
**(B)** The expression and prognostic value of miRNAs in ccRCC.
**(C)** Upstream lncRNAs that could potentially bind to let‐7e‐5p, miR‐204‐5p, miR‐27b‐3p and STEAP3. The red circle represents positive correlation, the blue circle represents negative correlation, and the size of the circle represents the absolute value of the correlation coefficient, the larger the absolute value the larger the circle.
**(D)** The expression and prognostic value of lncRNAs in ccRCC.

**Supplementary Table S1.** Abbreviations of cancers in the TCGA‐Pancancer cohort

**Supplementary Table S2.** The R packages that were used in this study

**Supplementary Table S3.** The statistical data of STEAP3 expression difference between tumor and normal tissues in pancancer

**Supplementary Table S4.** The expression of STEAP3 after immunotherapy

**Supplementary Table S5.** The relationships between STEAP3 expression and common chemotherapy agents and small molecular compounds.

## Data Availability

The unique contributions made in the work are described in the article as well as the Supplementary Material. Any further questions can be directed to the corresponding authors.

## References

[1] Siegel RL , Miller KD , Fuchs HE , Jemal A . Cancer statistics, 2022. CA Cancer J Clin. 2022;72(1):7‐33. 35020204 10.3322/caac.21708

[2] Ribas A , Wolchok JD . Cancer immunotherapy using checkpoint blockade. Science. 2018;359(6382):1350‐1355. 29567705 10.1126/science.aar4060 PMC7391259

[3] Crichton R . Iron Metabolism: from Molecular Mechanisms to Clinical Consequences. John Wiley & Sons; 2016.

[4] Ni S , Yuan Y , Song S , Li X . A double‐edged sword with a therapeutic target: iron and ferroptosis in immune regulation. Nutr Rev. 2022;81(5):587‐596. 10.1093/nutrit/nuac071 36130411

[5] Kim SH , Ho JN , Jin H , et al. Upregulated expression of BCL2, MCM7, and CCNE1 indicate cisplatin‐resistance in the set of two human bladder cancer cell lines: T24 cisplatin sensitive and T24R2 cisplatin resistant bladder cancer cell lines. Investig Clin Urol. 2016;57(1):63‐72. 10.4111/icu.2016.57.1.63 PMC4778756 26966728

[6] Tamura T , Chiba J . STEAP4 regulates focal adhesion kinase activation and CpG motifs within STEAP4 promoter region are frequently methylated in DU145, human androgen‐independent prostate cancer cells. Int J Mol Med. 2009;24(5):599‐604. 19787193 10.3892/ijmm_00000270

[7] Ohgami RS , Campagna DR , Greer EL , et al. Identification of a ferrireductase required for efficient transferrin‐dependent iron uptake in erythroid cells. Nat Genet. 2005;37(11):1264‐1269. 16227996 10.1038/ng1658 PMC2156108

[8] Ohgami RS , Campagna DR , McDonald A , Fleming MD . The Steap proteins are metalloreductases. Blood. 2006;108(4):1388‐1394. 16609065 10.1182/blood-2006-02-003681 PMC1785011

[9] Wang LL , Luo J , He ZH , et al. STEAP3 promotes cancer cell proliferation by facilitating nuclear trafficking of EGFR to enhance RAC1‐ERK‐STAT3 signaling in hepatocellular carcinoma. Cell Death Dis. 2021;12(11):1052. 34741044 10.1038/s41419-021-04329-9 PMC8571373

[10] Savci‐Heijink CD , Halfwerk H , Koster J , van de Vijver MJ . A novel gene expression signature for bone metastasis in breast carcinomas. Breast Cancer Res Treat. 2016;156(2):249‐259. 26965286 10.1007/s10549-016-3741-z PMC4819548

[11] Han M , Xu R , Wang S , et al. Six‐transmembrane epithelial antigen of prostate 3 predicts poor prognosis and promotes glioblastoma growth and invasion. Neoplasia. 2018;20(6):543‐554. 29730475 10.1016/j.neo.2018.04.002 PMC5994776

[12] Ye CL , Du Y , Yu X , et al. STEAP3 affects ferroptosis and progression of renal cell carcinoma through the p53/xCT pathway. Technol Cancer Res Treat. 2022;21:15330338221078728. 35275508 10.1177/15330338221078728 PMC8921746

[13] Machlenkin A , Paz A , Bar Haim E , et al. Human CTL epitopes prostatic acid phosphatase‐3 and six‐transmembrane epithelial antigen of prostate‐3 as candidates for prostate cancer immunotherapy. Cancer Res. 2005;65(14):6435‐6442. 16024648 10.1158/0008-5472.CAN-05-0133

[14] Weinstein JN , Collisson EA , Mills GB , et al. The cancer genome atlas pan‐cancer analysis project. Nat Genet. 2013;45(10):1113‐1120. 24071849 10.1038/ng.2764 PMC3919969

[15] Barretina J , Caponigro G , Stransky N , et al. The cancer cell line encyclopedia enables predictive modelling of anticancer drug sensitivity. Nature. 2012;483(7391):603‐607. 22460905 10.1038/nature11003 PMC3320027

[16] Jones J , Otu H , Spentzos D , et al. Gene signatures of progression and metastasis in renal cell cancer. Clin Cancer Res. 2005;11(16):5730‐5739. 16115910 10.1158/1078-0432.CCR-04-2225

[17] Digre A , Lindskog C . The human protein atlas‐spatial localization of the human proteome in health and disease. Protein Sci. 2021;30(1):218‐233. 33146890 10.1002/pro.3987 PMC7737765

[18] Ru B , Wong CN , Tong Y , et al. TISIDB: an integrated repository portal for tumor‐immune system interactions. Bioinformatics. 2019;35(20):4200‐4202. 30903160 10.1093/bioinformatics/btz210

[19] Gao J , Aksoy BA , Dogrusoz U , et al. Integrative analysis of complex cancer genomics and clinical profiles using the cBioPortal. Sci Signal. 2013;6(269):pl1. 23550210 10.1126/scisignal.2004088 PMC4160307

[20] McNamara MG , Jacobs T , Lamarca A , Hubner RA , Valle JW , Amir E . Impact of high tumor mutational burden in solid tumors and challenges for biomarker application. Cancer Treat Rev. 2020;89:102084. 32738738 10.1016/j.ctrv.2020.102084

[21] Dudley JC , Lin MT , Le DT , Eshleman JR . Microsatellite instability as a biomarker for PD‐1 blockade. Clin Cancer Res. 2016;22(4):813‐820. 26880610 10.1158/1078-0432.CCR-15-1678

[22] Zeng Z , Wong CJ , Yang L , et al. TISMO: syngeneic mouse tumor database to model tumor immunity and immunotherapy response. Nucleic Acids Res. 2022;50(D1):D1391‐d1397. 34534350 10.1093/nar/gkab804 PMC8728303

[23] Liu CJ , Hu FF , Xia MX , Han L , Zhang Q , Guo AY . GSCALite: a web server for gene set cancer analysis. Bioinformatics. 2018;34(21):3771‐3772. 29790900 10.1093/bioinformatics/bty411

[24] Szklarczyk D , Gable AL , Lyon D , et al. STRING v11: protein‐protein association networks with increased coverage, supporting functional discovery in genome‐wide experimental datasets. Nucleic Acids Res. 2019;47(D1):D607‐d613. 30476243 10.1093/nar/gky1131 PMC6323986

[25] Zhou Y , Zhou B , Pache L , et al. Metascape provides a biologist‐oriented resource for the analysis of systems‐level datasets. Nat Commun. 2019;10(1):1523. 30944313 10.1038/s41467-019-09234-6 PMC6447622

[26] Liberzon A , Birger C , Thorvaldsdóttir H , Ghandi M , Mesirov JP , Tamayo P . The molecular signatures database (MSigDB) hallmark gene set collection. Cell Syst. 2015;1(6):417‐425. 26771021 10.1016/j.cels.2015.12.004 PMC4707969

[27] Yuan H , Yan M , Zhang G , et al. CancerSEA: a cancer single‐cell state atlas. Nucleic Acids Res. 2019;47(D1):D900‐d908. 30329142 10.1093/nar/gky939 PMC6324047

[28] Li JH , Liu S , Zhou H , Qu LH , Yang JH . starBase v2.0: decoding miRNA‐ceRNA, miRNA‐ncRNA and protein‐RNA interaction networks from large‐scale CLIP‐Seq data. Nucleic Acids Res. 2014;42:D92‐D97. 24297251 10.1093/nar/gkt1248 PMC3964941

[29] Hamada K , Tsunoda T , Yoshimura K . Emerging immune‐monitoring system for immune checkpoint inhibitors. Life. 2022;12(8):1229. 36013407 10.3390/life12081229 PMC9410458

[30] Chen Z , Zhang D , Yue F , Zheng M , Kovacevic Z , Richardson DR . The iron chelators Dp44mT and DFO inhibit TGF‐β‐induced epithelial‐mesenchymal transition via up‐regulation of N‐Myc downstream‐regulated gene 1 (NDRG1). J Biol Chem. 2012;287(21):17016‐17028. 22453918 10.1074/jbc.M112.350470 PMC3366822

[31] Akatsuka S , Yamashita Y , Ohara H , et al. Fenton reaction induced cancer in wild type rats recapitulates genomic alterations observed in human cancer. PLoS One. 2012;7(8):e43403. 22952676 10.1371/journal.pone.0043403 PMC3430702

[32] Hjalgrim H , Edgren G , Rostgaard K , et al. Cancer incidence in blood transfusion recipients. J Natl Cancer Inst. 2007;99(24):1864‐1874. 18073377 10.1093/jnci/djm248

[33] Torti SV , Torti FM . Iron and cancer: more ore to be mined. Nat Rev Cancer. 2013;13(5):342‐355. 23594855 10.1038/nrc3495 PMC4036554

[34] Lui GY , Kovacevic Z , Richardson V , Merlot AM , Kalinowski DS , Richardson DR . Targeting cancer by binding iron: dissecting cellular signaling pathways. Oncotarget. 2015;6(22):18748‐18779. 26125440 10.18632/oncotarget.4349 PMC4662454

[35] Lane DJ , Mills TM , Shafie NH , et al. Expanding horizons in iron chelation and the treatment of cancer: role of iron in the regulation of ER stress and the epithelial‐mesenchymal transition. Biochim Biophys Acta. 2014;1845(2):166‐181. 24472573 10.1016/j.bbcan.2014.01.005

[36] Wang S , Chen L , Liu W . Matrix stiffness‐dependent STEAP3 coordinated with PD‐L2 identify tumor responding to sorafenib treatment in hepatocellular carcinoma. Cancer Cell Int. 2022;22(1):318. 36229881 10.1186/s12935-022-02634-7 PMC9563531

[37] Wu J , Bi Q , Zheng X , et al. STEAP3 can predict the prognosis and shape the tumor microenvironment of clear cell renal cell carcinoma. BMC Cancer. 2022;22(1):1204. 36424540 10.1186/s12885-022-10313-z PMC9686107

[38] Chanock SJ . The paradox of mutations and cancer. Science. 2018;362(6417):893‐894. 30467157 10.1126/science.aav5697

[39] Abu‐Hanna J , Patel JA , Anastasakis E , et al. Therapeutic potential of inhibiting histone 3 lysine 27 demethylases: a review of the literature. Clin Epigenetics. 2022;14(1):98. 35915507 10.1186/s13148-022-01305-8 PMC9344682

[40] Cheung CH , Coumar MS , Hsieh HP , Chang JY . Aurora kinase inhibitors in preclinical and clinical testing. Expert Opin Investig Drugs. 2009;18(4):379‐398. 10.1517/13543780902806392 19335272

[41] Yu Z , Wang H , Fang Y , et al. Molecular chaperone HspB2 inhibited pancreatic cancer cell proliferation via activating p53 downstream gene RPRM, BAI1, and TSAP6. J Cell Biochem. 2020;121(3):2318‐2329. 31692031 10.1002/jcb.29455

[42] Laskar S , Das R , Kundu S , et al. Whole exome sequencing identifies the potential role of genes involved in p53 pathway in nasopharyngeal carcinoma from Northeast India. Gene. 2022;812:146099. 34906645 10.1016/j.gene.2021.146099

[43] Ala U . Competing endogenous RNAs, non‐coding RNAs and diseases: an intertwined story. Cell. 2020;9(7):1574. 10.3390/cells9071574 PMC7407898 32605220

[44] Ciafrè SA , Russo M , Michienzi A , Galardi S . Long noncoding RNAs and cancer stem cells: dangerous liaisons managing cancer. Int J Mol Sci. 2023;24(3):24. 10.3390/ijms24031828 PMC9915130 36768150

[45] Kim WR , Park EG , Lee DH , Lee YJ , Bae WH , Kim HS . The tumorigenic role of circular RNA‐microRNA axis in cancer. Int J Mol Sci. 2023;24(3):3050. 36769372 10.3390/ijms24033050 PMC9917898

[46] Gao W , Wu Y , He X , et al. MicroRNA‐204‐5p inhibits invasion and metastasis of laryngeal squamous cell carcinoma by suppressing forkhead box C1. J Cancer. 2017;8(12):2356‐2368. 28819440 10.7150/jca.19470 PMC5560155

[47] Bao CH , Guo L . miR‐27b‐3p inhibits invasion, migration and epithelial‐mesenchymal transition in gastric cancer by targeting RUNX1 and activation of the hippo signaling pathway. Anti Cancer Agents Med Chem. 2022;22(5):864‐873. 10.2174/1871520621666210707095833 34238170

[48] Miao W , Li N , Gu B , Yi G , Su Z , Cheng H . MiR‐27b‐3p suppresses glioma development via targeting YAP1. Biochem Cell Biol. 2020;98(4):466‐473. 32567955 10.1139/bcb-2019-0300

[49] Wu J , Liu T , Sun L , Zhang S , Dong G . Long noncoding RNA SNHG4 promotes renal cell carcinoma tumorigenesis and invasion by acting as ceRNA to sponge miR‐204‐5p and upregulate RUNX2. Cancer Cell Int. 2020;20:514. 33088220 10.1186/s12935-020-01606-z PMC7574175

[50] Wang D , Zhu X , Siqin B , Ren C , Yi F . Long non‐coding RNA CYTOR modulates cancer progression through miR‐136‐5p/MAT2B axis in renal cell carcinoma. Toxicol Appl Pharmacol. 2022;447:116067. 35597301 10.1016/j.taap.2022.116067

[51] Lv C , Yu H , Wang K , et al. ENO2 promotes colorectal cancer metastasis by interacting with the LncRNA CYTOR and activating YAP1‐induced EMT. Cell. 2022;11(15):2363. 10.3390/cells11152363 PMC9367517 35954207

[52] Liu Y , Geng X . Long non‐coding RNA (lncRNA) CYTOR promotes hepatocellular carcinoma proliferation by targeting the microRNA‐125a‐5p/LASP1 axis. Bioengineered. 2022;13(2):3666‐3679. 35081873 10.1080/21655979.2021.2024328 PMC8974008

[53] Chang Y , Li N , Yuan W , Wang G , Wen J . LINC00997, a novel long noncoding RNA, contributes to metastasis via regulation of S100A11 in kidney renal clear cell carcinoma. Int J Biochem Cell Biol. 2019;116:105590. 31442606 10.1016/j.biocel.2019.105590

[54] Shi Z , Shen C , Yu C , et al. Long non‐coding RNA LINC00997 silencing inhibits the progression and metastasis of colorectal cancer by sponging miR‐512‐3p. Bioengineered. 2021;12(1):627‐639. 33570445 10.1080/21655979.2021.1882164 PMC8806252

