# Supplementary material for: Six‐transmembrane epithelial antigen of prostate 3 (STEAP3) is a potential prognostic biomarker in clear cell renal cell carcinoma that correlates with M2 macrophage infiltration and epithelial–mesenchymal
Source: Cancer Rep (Hoboken). 2023 Jun 21;6(8):e1824. doi: 10.1002/cnr2.1824 (PMC10432435; doi:10.1002/cnr2.1824)
Supplement: Supplementary file 1 — Supplementary Figure S1. Association between STEAP3 expression and prognosis in pan‐cancer. (A‐B) Kaplan–Meier overall survival curves of STEAP3 in ACC and KIRP. (C) The expression distribution of STEAP3 in the different clinical stages of HNSC, KIRP, LIHC, and LUSC. (D) The expression distribution of STEAP3 in the different pathological stages of HNSC, LGG, LIHC, PAAD, STAD, and USEC. Supplementary Figure 2. The lncRNA‐miRNA‐STEAP3 regulatory network in ccRCC. (A) Upstream miRNAs that could potentially bind to STEAP3. The red circle represents positive correlation, the blue circle represents negative correlation, and the size of the circle represents the absolute value of the correlation coefficient, the larger the absolute value the larger the circle. (B) The expression and prognostic value of miRNAs in ccRCC. (C) Upstream lncRNAs that could potentially bind to let‐7e‐5p, miR‐204‐5p, miR‐27b‐3p and STEAP3. The red circle represents positive correlation, the blue circle represents negative correlation, and the size of the circle represents the absolute value of the correlation coefficient, the larger the absolute value the larger the circle. (D) The expression and prognostic value of lncRNAs in ccRCC. [file CNR2_1824_Supplemenary Figure_2.pdf › CNR2_1824_Supplemenary Figure_2.pdf]

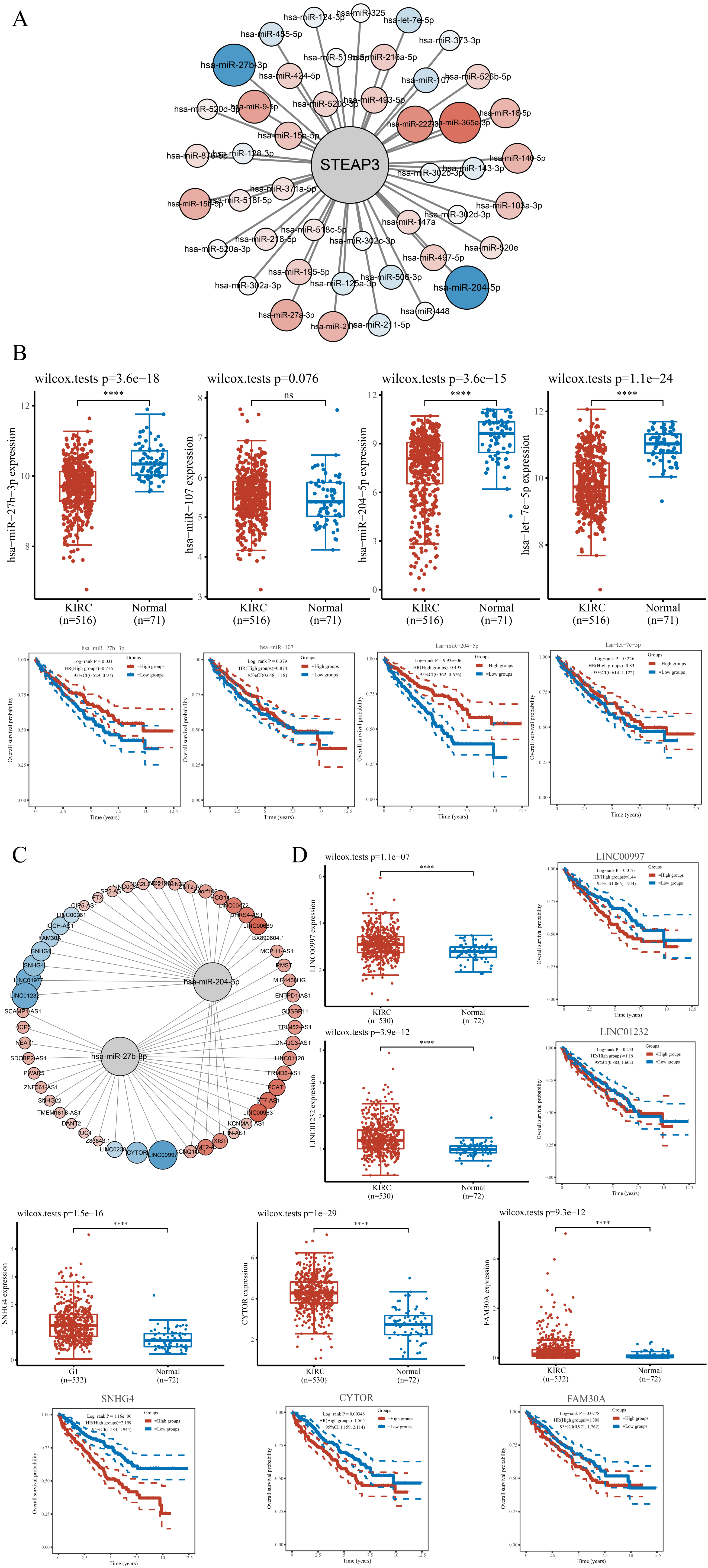
A
STEAP3
hsa-miR-27b-3p
hsa-miR-204-5p
hsa-miR-107
hsa-let-7e-5p
B
wilcox.tests p=3.6e−18
wilcox.tests p=0.076
wilcox.tests p=3.6e−15
wilcox.tests p=1.1e−24
hsa−miR−27b−3p expression
hsa−miR−107 expression
hsa−miR−204−5p expression
hsa−let−7e−5p expression
KIRC (n=516)
Normal (n=71)
Overall survival probability
Time (years)
C
hsa-miR-204-5p
hsa-miR-27b-3p
D
wilcox.tests p=1.1e−07
LINC00997 expression
KIRC (n=530)
Normal (n=72)
LINC00997
wilcox.tests p=3.9e−12
LINC01232 expression
LINC01232
wilcox.tests p=1.5e−16
SNHG4 expression
G1 (n=532)
wilcox.tests p=1e−29
CYTOR expression
wilcox.tests p=9.3e−12
FAM30A expression
KIRC (n=532)
SNHG4
CYTOR
FAM30A
